# Identifying Common Pathogenic Features in Deep Endometriotic Nodules and Uterine Adenomyosis

**DOI:** 10.3390/jcm10194585

**Published:** 2021-10-04

**Authors:** Christina Anna Stratopoulou, Alessandra Camboni, Jacques Donnez, Marie-Madeleine Dolmans

**Affiliations:** 1 Pôle de Recherche en Gynécologie, Institut de Recherche Expérimentale et Clinique, Université Catholique de Louvain, 1200 Brussels, Belgium; christina.stratopoulou@uclouvain.be (C.A.S.); alessandra.camboni@gmail.com (A.C.); 2 Anatomopathology Department, Cliniques Universitaires Saint-Luc, 1200 Brussels, Belgium; 3 Société de Recherche pour l’Infertilité, 1150 Brussels, Belgium; jacques.donnez@gmail.com; 4 Université Catholique de Louvain, 1200 Brussels, Belgium; 5 Gynecology Department, Cliniques Universitaires Saint-Luc, 1200 Brussels, Belgium

**Keywords:** endometriosis, deep endometriotic nodules, adenomyosis, platelets, macrophages, fibrosis, angiogenesis

## Abstract

Increasing imaging data point to a link between deep endometriotic nodules (DENs) and uterine adenomyosis (AD). The study aimed to investigate this link at the histological level and detect potential features shared by the two diseases. We collected formalin-fixed paraffin-embedded tissue (endometrium and lesions) from women with DENs of the rectovaginal septum (*n* = 13), AD (*n* = 14), and control subjects (*n* = 14). Immunohistochemical analyses of CD41 and CD68 were conducted to explore the roles of platelets and macrophages, respectively. Picrosirius red staining was carried out to gather evidence of fibrosis. Vascular endothelial growth factor (VEGF) was assessed, and total numbers of CD31-positive vessels were calculated to investigate the mechanism governing angiogenesis. Double immunohistochemistry for CD31 and alpha smooth muscle actin (αSMA) was performed to discern stable vessels. Platelet aggregation was significantly decreased in both types of lesions compared to their corresponding eutopic endometrium and healthy controls. Macrophage numbers were higher in both lesions than in their corresponding endometrium and healthy subjects. Significantly higher rates of collagen accumulation were detected in DENs and AD lesions compared to their corresponding eutopic and healthy endometrium. VEGF expression was downregulated in the stromal compartment of AD lesions compared to the healthy endometrium. The total number of vessels per area was significantly higher in DENs and AD lesions than in the healthy endometrium. Rates of αSMA-surrounded vessels were decreased in DENs and AD lesions compared to their corresponding eutopic and healthy endometrium. We report common pathogenic mechanisms between DENs and AD, namely excessive macrophage accumulation, fibrosis, and irregular angiogenesis. Our results further support the notion of DENs and AD being linked at the histological level.

## 1. Introduction

Endometriosis is a benign disease histologically characterized by the presence of endometrium-like tissue outside the uterus, typically inside the pelvic peritoneum, the ovaries, or the rectovaginal septum [1]. It is the cause of chronic pelvic pain, abnormal menstrual bleeding, and infertility, diminishing quality of life in up to 10% of the female population worldwide [2,3]. Adenomyosis (AD) involves development of endometrial tissue inside the myometrium and is frequently related to endometriosis, sharing many of the same symptoms [4,5,6,7]. As with endometriosis, AD has a high prevalence, estimated to be around 20% among reproductive-age women presenting with gynecological issues [8].

Deep endometriotic nodules (DENs) of the rectovaginal septum show morphological patterns distinct from peritoneal and ovarian types but resembling those of AD [1,6]. Despite long being recognized as separate entities, growing evidence from magnetic resonance imaging (MRI) and ultrasound demonstrates a link between DENs and AD and potentially a common origin [4,6,9]. It has indeed been hypothesized that DENs of the rectovaginal septum and AD might actually be alternative manifestations of the same disease rather than two distinct entities [6].

Increasing evidence points to the involvement of epithelial–mesenchymal transition (EMT) in the invasion process of endometriotic and adenomyotic lesions [7,10,11]. Accordingly, platelets and macrophages were both proposed as key mediators of EMT and fibroblast-myofibroblast transdifferentiation (FMT), eventually leading to endometrial invasiveness and fibrosis, respectively [12,13]. To assess the role of these processes in DENs and AD, we examined aggregation patterns of the two cell populations as well as evidence of collagen accumulation.

Upon invasion and lesion establishment, vascularization is pivotal to maintaining high metabolic activity in tissue. Endometrium has the unique ability to restore its vasculature periodically via neoangiogenesis, but this process may be enhanced or dysregulated in lesions [7,12]. Elevated expression of vascular endothelial growth factor (VEGF), a potent mediator of endothelial cell proliferation in the endometrium, was reported to contribute to this process, thus we conducted immunohistochemistry (IHC) to investigate its stromal and epithelial expression [14]. Given the capacity of endometrial tissue to form new vessels, we also assessed ectopic and eutopic microvessel density (MVD), a technique regularly used to predict the extent of angiogenesis in tissue samples of the same origin. Finally, we examined rates of vessels surrounded by alpha smooth muscle actin (αSMA)-positive cells in an attempt to assess the quality and the maturity of vessels in the different groups. Indeed, imaging data increasingly suggest a link between DENs and AD, and we now hypothesize that the two diseases also share pathogenic characteristics. The present study therefore aimed to identify patterns of modifications potentially common to both diseases at the histological level.

## 2. Materials and Methods

### 2.1. Study Participants and Tissue Collection

Samples were recovered from the anatomopathology archives of the Cliniques Universitaires Saint-Luc as formalin-fixed paraffin-embedded tissue blocks. DENs located in the rectovaginal septum in direct contact with the cervix and their corresponding endometrium were collected from 13 patients undergoing nodule resection and endometrial biopsy (61.5%) or nodule resection and hysterectomy (38.5%). None of the patients showed any signs of AD according to pelvic MRI images, and there were no myometrial lesions in histological specimens derived from hysterectomy. AD lesions and their corresponding endometrium were retrieved from 14 patients undergoing hysterectomy for symptomatic AD. Diagnoses were made by MRI and further confirmed by histological examination of surgical samples (collaboration with the Anatomopathology Department, Prof. E. Marbaix). MRI revealed diffuse AD in all cases, characterized by the presence of multiple adenomyotic foci and a thickened junctional zone (JZ) [15], while no signs of endometriosis were observed. From a histological point of view, ectopic endometrial tissue located at a depth of at least 2.5 mm inside the myometrium was identified as AD [16]. Control endometrial tissue was collected from 14 patients without endometrial pathology undergoing curettage (42.8%), hysteroscopic biopsy (28.6%), or hysterectomy (28.6%). Curettage and hysteroscopic biopsy were done for diagnostic purposes, mainly in the context of infertility, while hysterectomy was carried out for intramural myomas. Histological examination of the surgical specimens (collaboration with the Anatomopathology Department, Prof. E. Marbaix) confirmed the presence of normal endometrium in phase with the menstrual cycle. All recruited women were premenopausal with regular menstrual cycles, and none had taken hormone therapy for at least three months prior to surgery.

### 2.2. Immunohistochemistry

Platelets and macrophages were immunostained against specific surface receptors, namely CD41 and CD68, respectively. VEGF was selected as an important mediator of endothelial cell proliferation in endometrium. CD31 was used for detection of vessel endothelium and perivascular αSMA as a marker of mature stable vessels.

Serial 5 µm sections were cut from each paraffin block using a microtome. Upon deparaffinization and rehydration, they were incubated for 30 min in H_2_O_2_ solution to inhibit endogenous peroxidase. For antigen retrieval, the slides were heated to 98 °C in citrate buffer (pH = 6) for 75 min and then cooled to room temperature. They were subsequently incubated at 4 °C overnight with the primary antibody against CD41 (1/250; Abcam ab134131), CD68 (1/400; Abcam ab955), VEGF (1/250; ThermoFisher MA5-12184), or CD31 (1/500; Abcam ab134168). The slides were rinsed and incubated with EnVision anti-rabbit (Agilent K4003) or anti-mouse (Agilent K4001) horseradish peroxidase-labeled polymer for 60 min. Bound antibody complexes were stained with diaminobenzidine before counterstaining with Mayer’s hematoxylin. Finally, the slides were routinely dehydrated and mounted. Representative images of positive and negative controls can be found in Supplementary Figure S1.

Tissue sections were digitized at 20× magnification using the SCN400 slide scanner (Leica Biosystems, Wetzlar, Germany). For IHC analysis, at least 10 serial images were captured for each sample to display all available tissue from one side to the other. Stroma was encircled manually and analyzed for CD41 and CD68 immunostaining, while stroma and epithelium were surrounded and analyzed separately for VEGF. Positive expression was quantified as the percentage of area stained with diaminobenzidine color deconvolution per total surrounded area in pixels using a custom-made macro for open source Fiji-ImageJ software [17].

Vessels were stained against the endothelial cell marker CD31, and MVD was calculated based on a previously reported protocol with some modifications [18]. Briefly, at least 10 images were captured for each sample, and each image was divided into grid points of 450 µm^2^ in area. Total numbers of grid points hitting stromal tissue and vessels (small and large) immunostained against CD31 were counted for each image. MVD was calculated for each sample using the following formula:

MVD = ∑Ni/∑Vi

where ∑Ni = total number of vessels and ∑Vi = total number of grid points hitting stromal tissue. Results are expressed as the mean number of vessels per mm^2^ of stromal area.

### 2.3. Double Immunohistochemistry

Anti-human CD31 antibody (1/500; Abcam ab134168) was used to stain vessel endothelium. The protocol for immunostaining with the first primary antibody was the same as described in the previous section up to the substrate coloration step. The slides were then incubated at 4 °C overnight with the second primary antibody against human αSMA (1/400; Dako M0851). They were subsequently rinsed and incubated with secondary antibody (1/300; Jackson Immuno 115-055-166). The second substrate was stained with fast red (Sigma F4648) prior to counterstaining, dehydration, and mounting. Representative images of positive and negative controls can be seen in Supplementary Figure S1.

For analysis, the slides were scanned, and magnified images were obtained as previously described. Total vessel numbers identified by endothelial CD31 positivity were quantified. Vessels surrounded by αSMA-positive cells considered to be mature and stable were also counted. Results are expressed as the percentage of mature vessels per total vessel number in each sample.

### 2.4. Picrosirius Red Staining

Picrosirius red staining was conducted to detect collagen fibers in ectopic and eutopic stroma. Following deparaffinization and rehydration, the slides were immersed in 1% phosphomolybdic acid solution for 2 min, rinsed in water, and then incubated in saturated aqueous picric acid solution containing 0.1% direct red 80 (Sigma 365548) for 2 h. After brief washing in 0.01 N HCl and an additional rinse in water, the slides were routinely dehydrated and mounted. Sections were digitized using the SCN400 slide scanner at 20× magnification.

For analysis, at least 10 serial images (20×) were captured from each slide to display all available tissue from one side to the other. Stroma was outlined manually and analyzed for collagen fiber content (stained red) out of stromal area using a custom-made macro for Fiji-ImageJ [17].

## 3. Results

### 3.1. Clinicopathological Data

Characteristics of patients and controls are detailed in Table 1. Subjects in the control group, the DEN group, and the AD group were all comparable in terms of age and parity. The majority of AD patients (85.7%) presented with menorrhagia, a significantly higher rate than DEN patients (30.8%) (** *p* = 0.006). Dysmenorrhea rates were significantly higher in DEN (61.5%) and AD (57.1%) patients than in healthy subjects (14.3%) (* *p* = 0.018, 0.046).

### 3.2. Platelet Aggregation

**Figure 1:**
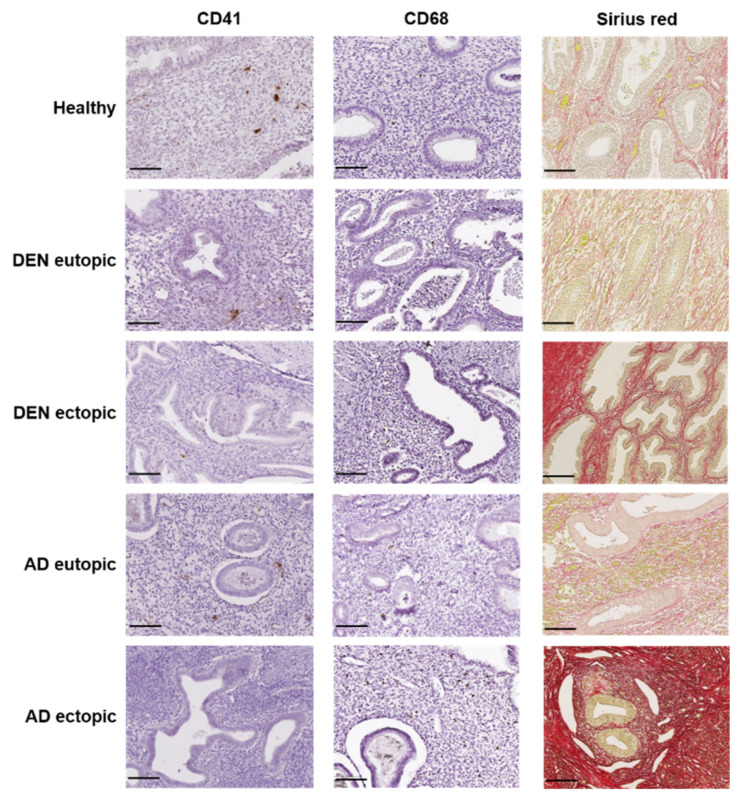
Representative images of platelet aggregation, macrophage accumulation, and fibrosis in endometrium: healthy; DEN eutopic; DEN lesion; AD eutopic; AD lesion. Scale bar (in black) represents 100 μm.

**Figure 2:**
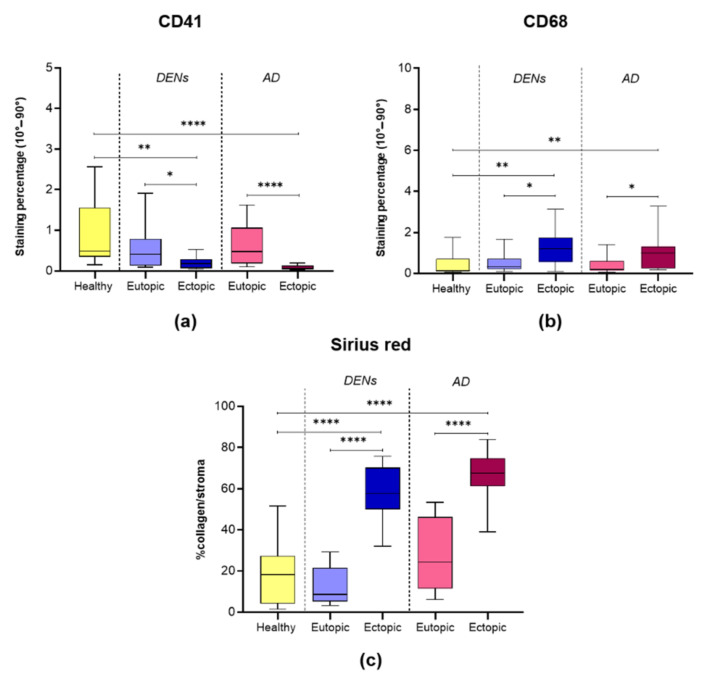
Boxplots showing levels of: (**a**) CD41 (* *p* = 0.028, ** *p* = 0.001, **** *p* < 0.0001); (**b**) CD68 (* *p* = 0.03, 0.038, ** *p* = 0.008, 0.004); (**c**) collagen fibers (**** *p* < 0.0001).

Aggregation of platelets, as indicated by CD41 IHC, was low in all groups and significantly decreased in cases of DENs and AD lesions compared to both their corresponding endometrium (* *p* = 0.028; **** *p* < 0.0001) and healthy controls (** *p* = 0.001; **** *p* < 0.0001) (Figure 1 and Figure 2a). No difference was encountered between eutopic endometrium from the two disease groups and the healthy controls.

### 3.3. Macrophage Accumulation

CD68-positive macrophages were spotted in all endometrial samples in varying numbers (Figure 1). Macrophage accumulation was significantly increased in DENs and AD lesions compared to their corresponding endometrium (* *p* = 0.037; 0.03) and healthy controls (Figure 1 and Figure 2b). CD68 staining levels were similar in eutopic endometrium from DEN and AD patients and healthy subjects.

### 3.4. Collagen Fiber Content

By optical microscopy, collagen fibers appeared red, while epithelium, blood, and other cells stained yellow (Figure 1). Analysis of picrosirius red staining demonstrated similarly low collagen content in DEN eutopic, AD eutopic, and control endometrium. On the contrary, collagen accumulation was evident in DENs and AD lesions and significantly higher than in their corresponding endometrium (**** *p* < 0.0001) and control tissue (**** *p* < 0.0001) (Figure 1 and Figure 2c).

### 3.5. Angiogenesis and Vessel Stability

**Figure 3:**
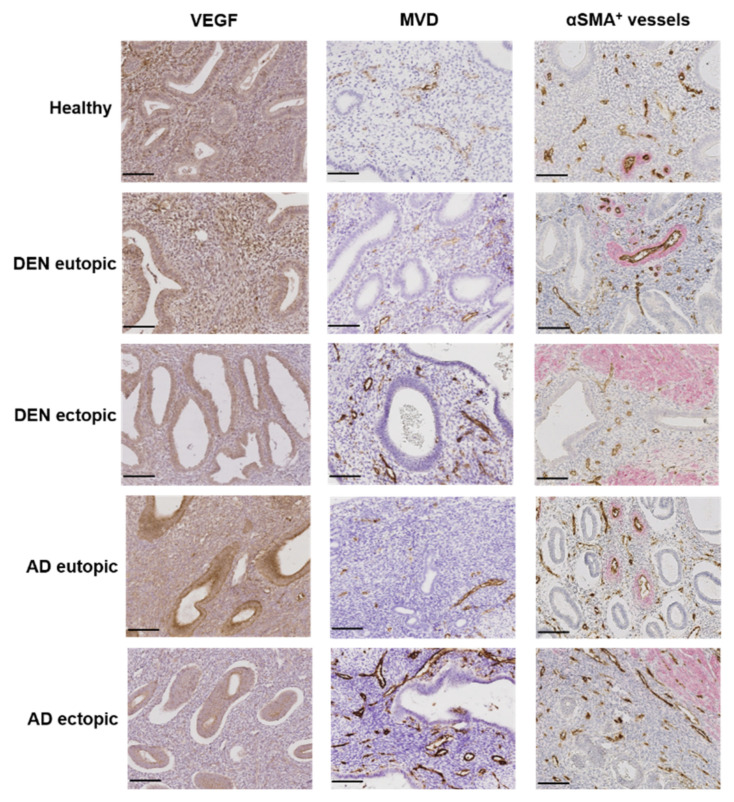
Representative images of VEGF expression, MVD, and presence of αSMA+ cells around vessels in endometrium: healthy; DEN eutopic; DEN lesion; AD eutopic; AD lesion. Scale bar (in black) represents 100 μm.

**Figure 4:**
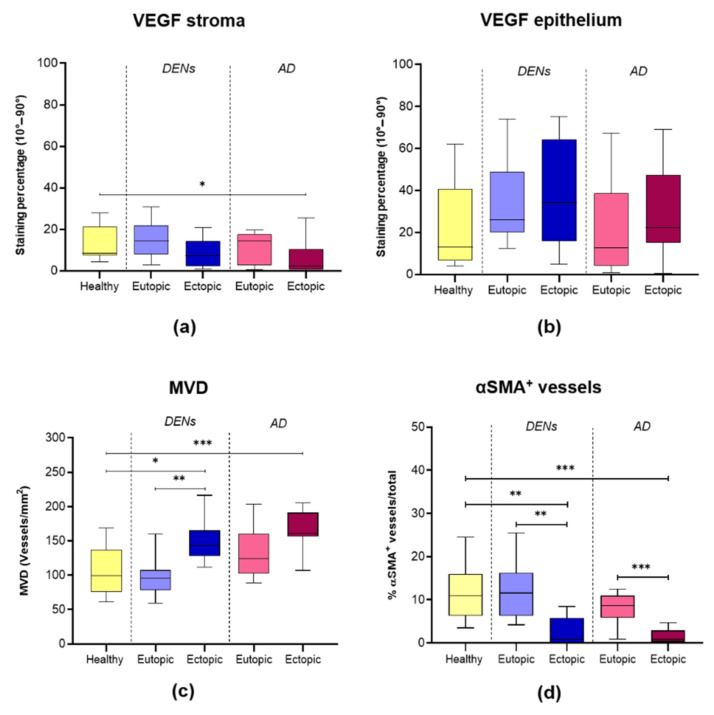
Boxplots showing levels of: (**a**) VEGF expression in stroma (* *p* = 0.022); (**b**) VEGF expression in epithelium; (**c**) MVD (* *p* = 0.013, ** *p* = 0.002, *** *p* = 0.0008); (**d**) αSMA+ vessels (** *p* = 0.002, 0.001; *** *p* = 0.0004, 0.0002).

The stromal compartment, the glands, and the vessel endothelium were all positive for VEGF (Figure 3). VEGF immunostaining showed significantly lower expression in the stroma of AD lesions than in healthy endometrium (* *p* = 0.022) (Figure 3 and Figure 4a). No other significant differences were recorded in either the stromal or the epithelial compartments of any of the three groups (Figure 4a,b).

MVD was significantly higher in DENs and AD lesions than in normal endometrium from healthy controls (* *p* = 0.013; *** *p* = 0.0008) (Figure 3 and Figure 4c). In DENs, ectopic endometrium exhibited significantly higher MVD than eutopic endometrium (** *p* = 0.002) (Figure 3 and Figure 4c). In AD, MVD was also greater in ectopic compared to eutopic endometrium but did not reach statistical significance (*p* = 0.07). MVD was found to be similar between healthy and eutopic endometrium from DEN and AD patients. Double IHC for CD31 and αSMA revealed the presence of αSMA-positive cells surrounding several vessels, forming one or more layers. Rates of vessels surrounded by αSMA-positive cells out of total vessels were significantly lower in DENs and AD lesions than in their corresponding eutopic (** *p* = 0.001; *** *p* = 0.0002) and healthy (** *p* = 0.002; *** *p* = 0.0004) tissues (Figure 3 and Figure 4d). No difference was observed between eutopic endometrium from the DEN and the AD groups and the control tissue.

## 4. Discussion

By comparing DENs and AD side by side, we identified specific pathogenic features shared by the two diseases, namely a drop in platelet numbers, excess macrophage accumulation, fibrosis, and irregular angiogenesis. The notion of DENs and AD being linked has been actively supported for a number of years now, primarily based on MRI and ultrasound data showing remarkably high rates of coexistence [4,6,9,19]. A popular hypothesis on the origin of AD suggests that ectopic lesions derive from the endometrial basalis by direct invasion of the myometrium via a traumatized JZ with subsequent progression and proliferation [4,5,6]. An abundance of experimental evidence also supports the role of lesion invasiveness in both DENs and AD [10,11,20,21]. To explain this invasive capacity of endometrial cells, EMT was reported to occur in endometriosis and AD [10,11]. This process is characterized by loss of cell polarity and destabilization of tight cell-to-cell junctions, eventually leading to transformation to mesenchymal cells with invasive capacity and, simultaneously, to FMT [22]. FMT, a closely related process, is defined as transition of fibroblasts to collagen-producing myofibroblasts, resulting in extracellular matrix accumulation and fibrosis [22]. Consistent with this theory, we found evidence of fibrosis in both DENs and AD lesions, where thick collagen fibers were shown to accumulate.

Platelets have been blamed for triggering EMT and FMT in AD via various growth factors they secrete [12]. In our previous study, however, we failed to confirm platelet aggregation in AD lesions and only observed minimal immunostaining for CD41 [23]. We now note the same tendency in both DENs and AD and, more specifically, significantly lower platelet aggregation in lesions compared to their respective eutopic and healthy endometrium. Once again, our results do not point to a fundamental role for platelets in DEN and AD pathogeneses.

On the other hand, we detected an increase in macrophage accumulation in DENs and AD lesions, exceeding levels in their respective eutopic and control endometrium. It is known that excessive macrophage accumulation and related inflammation can result in disease invasion and fibrosis, thus it is likely that they also mediate these processes in DENs and AD [13,24]. In particular, it was suggested that macrophages are the underlying cause of endometriosis, as they misinterpret ectopic endometrial tissue as a wound that they constantly try to repair [25]. The abundance of macrophage-secreted cytokines and growth factors may then activate EMT and FMT, eventually giving rise to lesion invasiveness and fibrosis [24].

At the same time, lesion-resident macrophages appear to possess neurogenic and nerve-sensitizing activity, thus their presence may explain the high dysmenorrhea rates found in DEN and AD patients in this study [26]. DENs are notorious for causing dysmenorrhea, with nodule resection yielding considerable alleviation of pain symptoms [27]. For this reason, lesion innervation has been extensively researched over the years, and evidence deriving from both the baboon model of endometriosis and human biopsies supports its role in lesions and pain induction [20,21,27]. Moreover, DEN nerves appear to be encapsulated in fibrotic tissue, especially in patients experiencing more adverse symptoms [27]. High rates of dysmenorrhea recorded in DEN and AD patients involved in this study combined with concurrent fibrosis and excess macrophage accumulation detected in lesions indicate a role and possible interaction of these factors during neurogenesis, leading to pain symptoms.

As with normal endometrium, ectopic lesions undergo cyclic bleeding, hence, it is logical that a mechanism of repair and vessel regeneration also resides in this tissue [28]. We detected higher numbers of vessels per stromal area in both types of lesions than in healthy endometrium, in line with the notion of aberrant angiogenesis in DENs and AD. On the contrary, we could not confirm an upturn in VEGF, once again suggesting an alternative means of angiogenesis [23]. It is known that VEGF expression in endometrium is regulated by hypoxia, which explains why it is increased during and after menstruation as it attempts to regenerate lost vasculature [14,29]. Upon endometrial repair, newly formed vessels seek to restore oxygen levels, leading to a decrease in VEGF. It is therefore likely that the greater number of vessels found in lesions accounts for the decrease in stromal VEGF expression by preventing hypoxia and subsequent signaling pathway activation. Nevertheless, the complex interplay between hypoxia, VEGF, and angiogenesis during the different phases of the menstrual cycle requires more in-depth studies to draw robust conclusions.

Despite increased numbers of vessels observed in lesions, only limited numbers were positive for αSMA. The presence of αSMA in pericytes is common and signals physiological vessel maturation and function [30,31]. However, in pathological conditions such as certain malignancies, pericyte αSMA levels were found to be lower, possibly indicating neoangiogenesis and metastatic activity [32,33]. Indeed, decreased rates of αSMA-positive vessels in ectopic lesions in DENs and AD further corroborate the notion of disease progression and neoangiogenesis, with vessels not reaching full maturation. Lower VEGF expression and platelet numbers found in ectopic lesions may also contribute to defective vessels, given the importance of these parameters in vessel maturation and maintenance of vascular integrity [34]. All these results suggest that angiogenesis occurring in ectopic lesions is irregular and/or defective, differing from physiological cyclic angiogenesis in endometrium.

Although endometriosis and uterine AD are recognized as separate diseases, an association between DENs and AD has found strong support. More than 20 years ago, it was argued that superficial peritoneal endometriosis, ovarian endometriomas, and DENs should be considered distinct entities from morphological and pathogenic perspectives, with the latter resembling AD lesions [1]. Our current study provides new evidence endorsing the notion that DENs and AD lesions are alike morphologically and have similar pathogenic features but, at the same time, differ substantially from their corresponding eutopic endometrium. Hence, the concept of retrograde menstruation alone accounting for DEN and AD development is highly unlikely. We believe that a more complex process is in play during lesion development. It is possible that certain endometrial cells acquire invasive capabilities and escape the JZ to generate AD lesions and, subsequently, DENs. Alternatively, Müllerian remnants displaced during embryonic development or adult progenitor cells may also be involved in de novo generation of endometrial tissue in ectopic locations [5,7]. We suspect that AD lesions may actually induce DEN development by proliferating and progressing to extrauterine locations [4,6]. More specifically, a hyperestrogenic environment has been described in both diseases and appears to play a pivotal role in disease initiation and evolution [3,5,7,35]. It is believed that hyperestrogenism and ensuing uterine hypercontractility result in disruption of the endometrial-myometrial JZ and trigger the tissue injury and repair mechanism [35]. Tissue-repairing leukocytes such as macrophages accumulate at the site of injury, then secrete numerous growth factors and cytokines in an attempt to heal the trauma, enhancing cell proliferation, neoangiogenesis, EMT, and FMT, thereby facilitating lesion progression and fibrotic tissue formation. [24,25]. Over time, lesions continue to proliferate and invade adjacent extrauterine tissue, generating DENs [4,6].

A limitation of this study was that the retrospective nature of the data did not allow us to follow changes happening in real time, thus it was impossible to predict the exact stage at which the events we observed took place. Indeed, angiogenesis is known to occur periodically in endometrium and ectopic lesions, but it is not known if it was going on at the time of sample collection. On the other hand, DENs and AD are two very slowly evolving entities, but, as previously stressed, nobody is born with stage IV endometriosis [36]. In other words, in order for DENs to evolve from C1 (≤1 cm) to C3 (≥3 cm) according to the #Enzian classification [37], we must accept that, at a certain point in time, they possess the capacity to grow and progress [36,38]. Our goal here was to detect similarities between these two gradually developing diseases, hoping to pave the way for more extensive research into their potential common origin.

The key to optimal diagnosis and clinical management of endometriosis and AD is in-depth understanding of their pathophysiology and underlying causes. It is becoming increasingly evident that endometriosis is, in fact, a multifactorial systemic disease rather than simple growth of ectopic endometrial tissue somewhere inside the pelvic cavity [3]. AD stills troubles clinicians with regard to diagnostic techniques and use of a universally accepted classification system and, as a result, sometimes remains undiagnosed until the postoperative stage [5,15,39]. AD and endometriosis, especially DENs, frequently coexist and are most likely linked, as indicated by imaging data and the common pathogenic features we identified [4,6,9]. In studies by Donnez et al. [6] and Chapron et al. [9], focal AD of the outer myometrium (FAOM) significantly correlated with the presence of DENs. In this study, we selected “pure” cases (namely only DENs or only AD) to avoid bias when comparing the two types of lesions, but similarities appear to exist even in these cases. It was argued that FAOM may have a pathogenesis distinct from diffuse adenomyosis but common to endometriosis [9,40]. Experts in the field proposed the possibility of endometriosis and AD actually being two phenotypes of the same disease, explaining their multiple common characteristics [6,41,42]. Consistent with this theory, we showed that diffuse adenomyotic lesions also share some common features with DENs. AD may actually be the underlying cause of symptom recurrence following DEN resection, stressing the critical importance of considering the link between these two diseases in routine clinical practice [19]. The role of estrogen should be emphasized, as it impacts both conditions and may drive their pathogeneses and related symptoms [3,5,35,42,43]. Indeed, innovative treatment options aiming to reduce estrogen levels appear to attenuate symptoms in severe cases of both endometriosis and AD [3,42,43,44,45]. It is nevertheless clear that multiple other factors are involved in the two pathogeneses, and we were able to identify some of them. To our knowledge, this is the first study to reveal specific common pathogenic characteristics of rectovaginal DENs and AD and provide evidence of their association at the histological level.

## 5. Conclusions

We confirm the existence of common pathogenic features between DENs and AD at the histological level, namely a decrease in platelet numbers, macrophage accumulation, fibrosis, and irregular angiogenesis. Our study points to a potential common pathogenesis for these diseases, providing the springboard to delve deeper into the implications of our findings.

## Figures and Tables

**Table 1:** Clinicopathological characteristics of patients and healthy controls involved in the study. AD patients showed significantly higher rates of menorrhagia than DEN patients (** *p* = 0.006). Rates of dysmenorrhea were found to be significantly higher in both DEN and AD patients than in healthy controls (* *p* = 0.018; 0.046).

Item	Healthy (a)	Endometriosis (b)	Adenomyosis (c)	*p*-Value
Age	Mean = 41.7	Mean = 38.7	Mean = 45.9	
	SD = 8.4	SD = 8.2	SD = 4.8	NS
	Range = 19–56	Range = 23–51	Range = 34–54	
Menstrual Phase Proliferative Secretory Menstrual	4 5 5	6 6 1	5 5 4	
Parity	Mean = 2	Mean = 1	Mean = 1.5	
	SD = 1.6	SD = 0.9	SD = 1.2	NS
	Range = 0–4	Range = 0–2	Range = 1–4	
Menorrhagia (%)	NE	30.8	85.7	(a, b) = NS (a, c) = NS ** (b, c) = 0.006
Dysmenorrhea (%)	14.3	61.5	57.1	* (a, b) = 0.018 * (a, c) = 0.046 (b, c) = NS

SD: standard deviation; NS: not significant; NE: not evaluated due to missing data.

## Data Availability

Data will be shared on reasonable request to the corresponding author.

## Supplementary Materials

The following are available online at https://www.mdpi.com/article/10.3390/jcm10194585/s1, Figure S1: Representative images of positive and negative controls.
