# Supplementary material for: Identifying Common Pathogenic Features in Deep Endometriotic Nodules and Uterine Adenomyosis
Source: J Clin Med. 2021 Oct 4;10(19):4585. doi: 10.3390/jcm10194585 (PMC8509556; doi:10.3390/jcm10194585)
Supplement: Supplementary file 1 [file jcm-10-04585-s001.zip › jcm-1387608-supplementary.pdf]

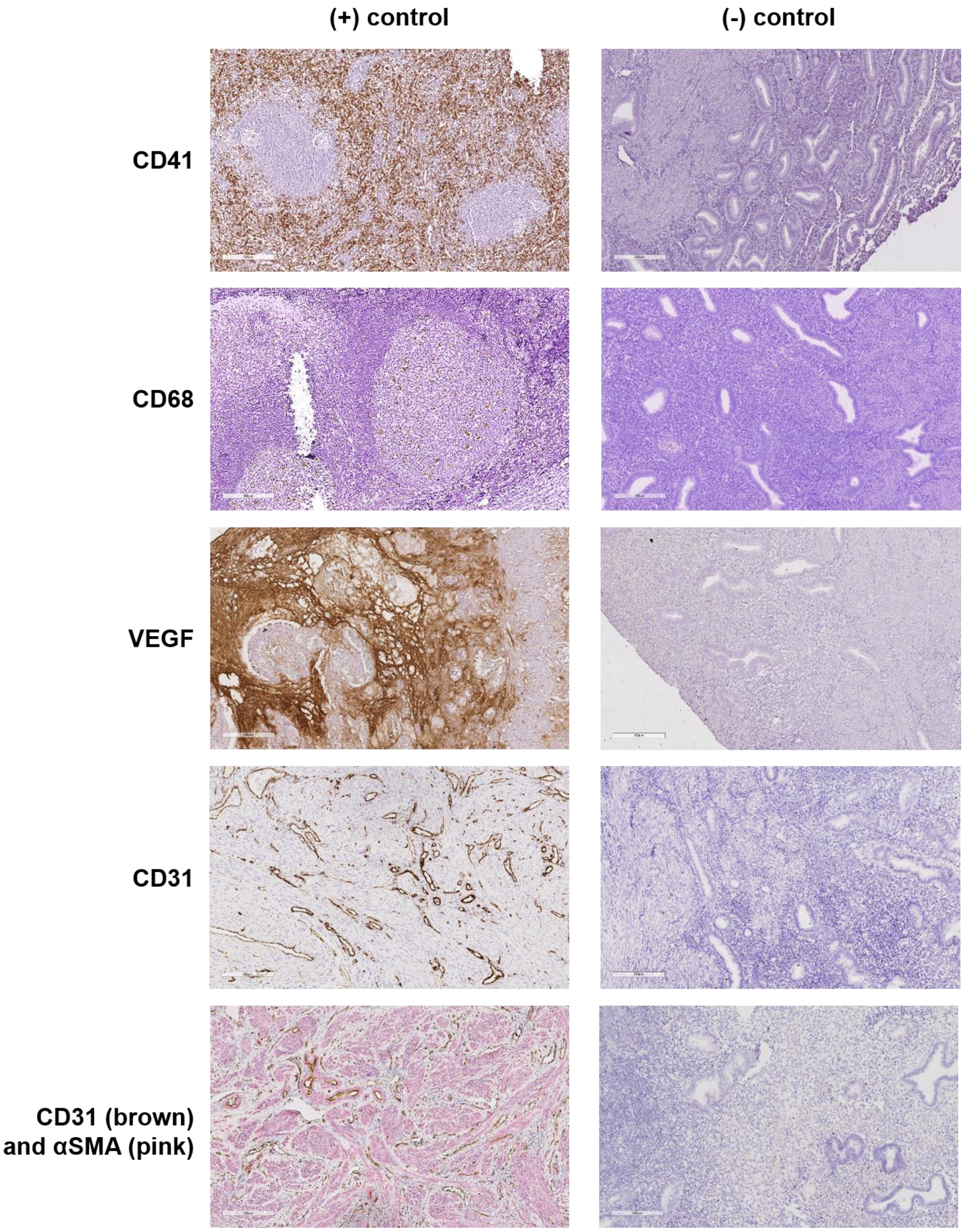

Supplementary figure S1. Representative images of positive and negative controls. As positive control we used human tissue sections from: spleen for CD41; tonsil for CD68; rectum for VEGF; uterus for CD31; uterus (smooth muscle) for CD31+αSMA. Endometrial sections were used as negative control. Scale bar represents 200 µm.
